# Tacrolimus Modulates TGF-β Signaling to Induce Epithelial-Mesenchymal Transition in Human Renal Proximal Tubule Epithelial Cells

**DOI:** 10.3390/jcm5050050

**Published:** 2016-04-26

**Authors:** Jason Bennett, Hilary Cassidy, Craig Slattery, Michael P. Ryan, Tara McMorrow

**Affiliations:** 1 Centre for Cell Signaling and Inflammation, Department of Medicine, Imperial College London, Hammersmith Hospital Campus, Du Cane Road, London W12 0NN, UK; j.bennett@imperial.ac.uk; 2 Renal Disease Research Group, School of Biomolecular and Biomedical Science, UCD Conway Institute, University College Dublin, Dublin 4, Ireland; hilary.cassidy@ucdconnect.ie (H.C.); craig.slattery@ucd.ie (C.S.); Michael.P.Ryan@ucd.ie (M.P.R.)

**Keywords:** Epithelial-mesenchymal transition, tacrolimus, fibrosis

## Abstract

Epithelial-mesenchymal transition (EMT), a process which describes the trans-differentiation of epithelial cells into motile mesenchymal cells, is pivotal in stem cell behavior, development and wound healing, as well as contributing to disease processes including fibrosis and cancer progression. Maintenance immunosuppression with calcineurin inhibitors (CNIs) has become routine management for renal transplant patient, but unfortunately the nephrotoxicity of these drugs has been well documented. HK-2 cells were exposed to Tacrolimus (FK506) and EMT markers were assessed by RT PCR and western blot. FK506 effects on TGF-β mRNA were assessed by RT PCR and TGF-β secretion was measured by ELISA. The impact of increased TGF-β secretion on Smad signaling pathways was investigated. The impact of inhibition of TGF-β signaling on EMT processes was assessed by scratch-wound assay. The results presented in this study suggest that FK506 initiates EMT processes in the HK-2 cell line, with altered expression of epithelial and myofibroblast markers evident. Additionally, the study demonstrates that FK506 activation of the TGF-β/ SMAD pathways is an essential step in the EMT process. Overall the results demonstrate that EMT is heavily involved in renal fibrosis associated with CNI nephrotoxicity.

## 1. Introduction

Chronic kidney disease (CKD) has been estimated to effect up to 10% and 13% of the European and US populations respectively [1,2,3,4] and represents a significant financial burden on health care systems globally. CKD progression is hallmarked by a pathological accumulation of extracellular matrix (ECM) components leading to disruption of organ architecture and functional capacity, with an eventual progression to end stage renal disease (ERSD) and organ failure. Initiating factors for CKD leading to ESRD are varied and include age [5], hypertension [6], diabetes [7], obesity [8,9] and therapeutic drug toxicity [10].

The advent of immunosuppressive agents revolutionized the process of solid organ transplantation, improving the rate of patient and graft survival dramatically. However, despite obvious benefits, the use of immunosuppressive agents is associated with the onset of acute and chronic nephrotoxicity, limiting their clinical use [11,12,13]. Calcineurin inhibitor (CNI) nephrotoxicity has been well documented in transplant recipients [14]; virtually all individuals receiving a CNI will develop some degree of kidney toxicity [15]. CKD has been documented to develop frequently following non-renal solid-organ transplantation and is strongly linked to increased morbidity and mortality within these individuals [16].

**Figure 1:**
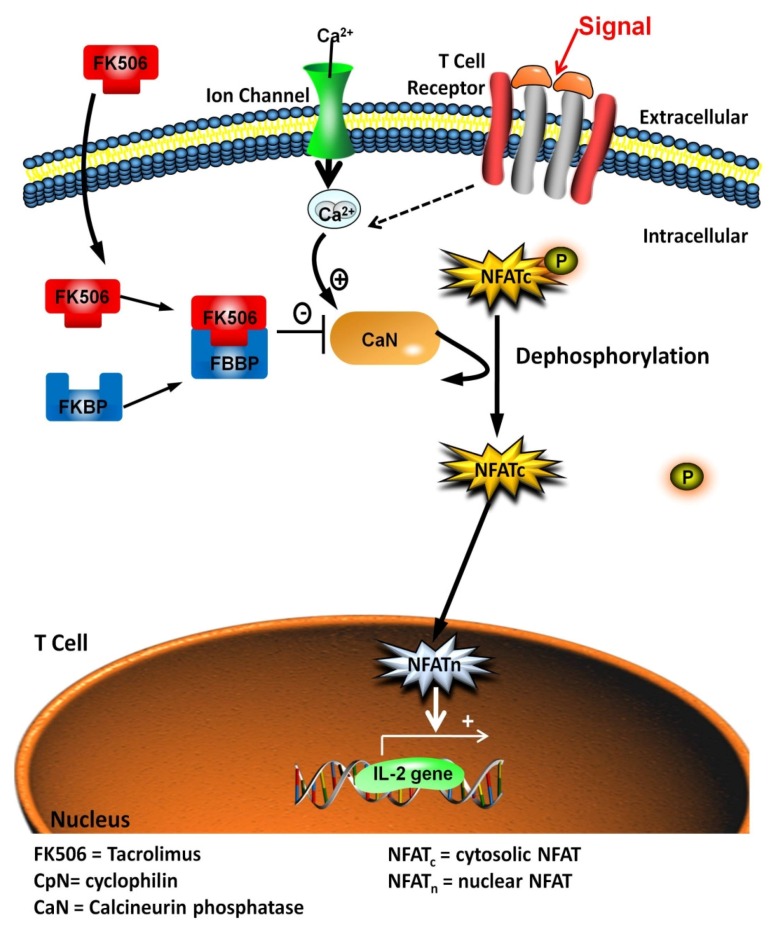
Mechanism of action of tacrolimus (FK506). In the cytoplasm tacrolimus (FK506) binds to the immunophilin FK506-binding protein (FKBP). The resulting complex then binds to the enzyme calcineurin (CaN), preventing the dephosphorylation of the cytoplasmic component of the nuclear factor of activated T-cells (NF-ATc). This blocks the transport of NF-ATc into the nucleus, preventing the binding of NF-ATn to the nuclear promoter of the interleukin-2 (IL-2) gene. Consequently, T cells do not produce IL-2, which is necessary for full T-cell activation.

Tacrolimus (FK506) has been employed as a primary and rescue immunusuppressive agent in the transplantation of all solid organs [17]. FK506, a CNI immunosuppressive agent, elicits its effects through complexing with the intracellular cytosolic immunophilin, FK506 binding protein 12 (FKBP12), which subsequently inhibits calcineurin phosphatase, thereby preventing the dephosphorylation of nuclear factor of activated T-cells (NFAT) family members responsible for the transcription of T-cell activating cytokines interleukin-2 and -4 [18] (Figure 1). Acute FK506 nephrotoxicity is reversible and causes a hemodynamic change characterized by renal vasoconstriction that is dose-dependent [19]. Chronic FK506-induced nephrotoxicity is irreversible and changes include tubular atrophy, afferent arteriolar hyalinosis and striped tubulointerstitial fibrosis (TIF) with mononuclear cell infiltration [20]. In order to safeguard future transplant patients and ease financial burden, it is of the utmost importance to increase our understanding of the mechanisms of FK506 nephrotoxicity in order to define new therapeutic strategies to ameliorate or avoid renal injury while maintaining adequate immunosuppression. Various studies suggest that FK506 is comparable to, if not better than, cyclosporine (CsA)-based therapy, in terms of patient and graft survival. Trials have compared CsA and FK506 in renal transplant recipients [21,22], with recent meta-analysis finding fewer acute rejection episodes and graft loss with FK506 [23]. A recent retrospective study indicated a more rapid decline in GFR in patients treated with CsA compared to FK506, although no difference was observed in mean arterial pressure, total cholesterol or the incidence of new-onset diabetes [24].

An optimal maintenance immunosuppression regime has not yet been established for transplantation, however, current trends are comparable between children, adolescents and adults. Over the course of the past 20 years a shift in the preferential immunosuppressant has become evident, with the percentage of adult patients discharged on CsA maintenance regimes declining from approximately 67% in 1998 to less than 4% in 2011 [25]. Conversely, FK506 has gained popularity as part of current immunosuppression regimes, with its use increasing in adult transplant recipients from approximately 26% in 1998 to 84% in 2011 [25]. This switch in preference is primarily based on emerging evidence related to FK506’s reduced nephrotoxicity in comparison to the once frequently prescribed CsA. In an effort to minimize the nephrotoxicity that has been reported with both of these CNIs more centres are favouring calcineurin minimization and withdrawal as opposed to complete avoidance. A landmark trial, the ELITE-Symphony trial, demonstrated better allograft outcomes at three years of follow-up in patients on low dose FK506 (in addition to steroids and mycophenolate mofetil) in comparison to standard or reduced doses of CsA or low dose sirolimus as the primary maintenance agent [26].

Of the two fibrotic lesions classically associated with CKD, glomerulosclerosis and TIF, the extent of TIF is consistently demonstrated to correlate with the rapid decline in renal function [27]. The primary cell type responsible for the excessive accumulation of ECM components during CKD progression is the myofibroblast, a mesenchymal cell possessing the phenotypic characteristics of fibroblasts and smooth muscle cells. While many theories have been proposed regarding the origins of myofibroblasts in diseased kidneys, accumulating evidence suggests that there is an inherent plasticity in the phenotype of cells; where once it was thought that cellular differentiation was a terminal state; it is now known that under the appropriate conditions some cell types have the ability to alter their phenotype through a process known as *trans*-differentiation.

Epithelial cells, originally considered to be terminally differentiated, are now known to be capable of altering their phenotype in response to morphogenic pressure from developmental cues or injured tissue, to a cell-type hallmarked by expression of mesenchymal markers, in a process referred to as epithelial-mesenchymal transition (EMT) which is a form of *trans*-differentiation. This transition involves the de-differentiation of epithelial cells, whereby the classical epithelial characteristics are lost, coupled with the acquisition of features specific to cells of a mesenchymal phenotype. An increasing body of evidence suggests that this phenotypic transformation of proximal tubular epithelial cells (PTECs) into myofibroblasts acts as a contributing factor to the interstitial myofibroblast population.

Studies have demonstrated EMT in human renal disease biopsy samples from various etiologies through the identification of interstitial cells which co-stain for both epithelial and mesenchymal cell markers [28]. In this study, the number of PTECs expressing mesenchymal markers was shown to correlate with serum creatinine levels and the degree of interstitial damage. Taken together these results suggest a strong contributory role for renal EMT in disease progression. Consequently, tubular epithelial cells may no longer be regarded as passive victims, but as activate contributors to renal disease [29]. The main aim of the present study was to investigate the ability of chronic FK506 exposure to induce EMT in PTECs and elucidate potential signaling pathways activated in response to FK506.

## 2. Experimental Section

**Cell culture:** The HK-2 human renal proximal tubular epithelial cell line was obtained from the American Type Culture Collection (Manassas, VA, USA). RPTEC cells were cultured in Dulbecco’s Modified Eagles Medium (low glucose)/Nutrient Mix F12 (DMEM/F12) containing 5 μg/mL insulin, 5 μg/mL transferrin, 5 ng/mL selenium, 36 ng/mL hydrocortisone, 4 pg/mL triiodo-1-thyronine, 10 ng/mL epidermal growth factor, 50 U/mL penicillin, 50 μg/mL streptomycin and 2 mM glutamine. Cell culture media was changed every 48 h. Cells were maintained in 75 cm^2^ Costar flasks at 37 °C in a humidified atmosphere containing 95% air and 5% CO_2_. Cells used in the inhibitor studies were pre-treated with SD-208 for 1 h, prior to incubation with FK506.

**Cell treatment:** FK506 (Alexis Biochemicals, Lausanne, Switzerland) was prepared as a 1 mM stock solution by dissolving 1 mg of the powder in 1.243 mL of 100% ethanol. Cyclosporine A (CsA) (Sigma-Aldrich, St. Louis, MO, USA) was prepared as a 5 mM stock solution by dissolving 5 mg of the powder in 833.33 µL of 100% ethanol. TGF-β1 (PromoKine, Heidelberg, Germany) was prepared as a stock solution of 5 nM by dissolving 5 µg of the lyophilized powder in sterile ultra-pure water. SD-208, a TGF-β Type I receptor inhibitor V (Calbiochem, San Diego, CA, USA), was prepared as a 10 mM stock solution dissolved 2 mg of the powder in 566.9 µL of dimethylsulfoxide (DMSO) and protected from light. Cells used in the inhibitor studies were pre-treated with SD-208 for 1 h, prior to incubation with FK506. For experiments investigating FK506 and CsA only the vehicle control cells were treated with pre-warmed DMEM/F12 media containing 0.1% ethanol. For experiments investigating FK506, CsA and TGF-β1 vehicle control cells were treated with 0.1% DMSO/0.1% ethanol.

**Cell Morphology:** Phase contrast microscopy was carried out using a JVC high resolution digital camera (KY-F55BE; JVC, London, UK) attached to a Nikon TMS phase contrast microscope (Nikon, Surrey, UK). Micrographs were then processed using Adobe Photoshop^®^ (CS5 version 12.0) software.

**Cytomic Assays:** HK-2 cell viability was assessed using the resazurin reduction assay (Sigma-Aldrich, St. Louis, MO, USA). This assay was conducted according to the manufacturer’s protocol and the viability of the cells was expressed as a percentage of the absorbance recorded for control cells. Cytotoxicity was assessed using the lactate dehydrogenase (LDH) activity assay (Sigma-Aldrich, St. Louis, MO, USA). After the indicated treatments, 100 µL of cell supernatants were assayed for LDH activity according to the manufacturer’s instructions. Cell proliferation was analyzed using the bromodeoxyuridine (BrdU) cell proliferation assay (Calbiochem, San Diego, CA, USA) to quantify the incorporation of BrdU, a thymidine analog, into newly synthesized DNA.

**Scratch Wound Assay:** RPTEC cells were grown to confluency in 12-well plates. The cell monolayer was then “wounded” using a 1 mL pipette tip and washed twice with warm, sterile PBS. Cells were then incubated with stated reagent treatments. Images were taken using a phase contrast microscope at time points 0, 12 and 24 h. The scratch area was then analyzed using ImageJ (Version 1.49) (http://rsb.info.nih.gov/ij/) and results expressed as percentage wound closure relative to the area of the same scratch at time 0 h, which was expressed as 100% area, and correspondingly, 0% closure.

**Western blot analysis:** Total protein was isolated from HK-2 cells using the RIPA buffer method according to the manufacturer’s protocol (Sigma-Aldrich, St. Louis, MO, USA). Equal amounts of whole cell lysates were analyzed by SDS-PAGE as described by Laemmli [30]. Immunoblotting was performed with antibodies specific for E-cadherin (1:1000) and Vimentin (1:5000) (BD Biosciences, San Jose, CA, USA), Fibronectin (1:3000; Calbiochem, San Diego, CA, USA), GAPDH (1:10,000), phospho-Smad2 and Smad2 (1:1000) (New England Biolabs, Beverly, MA, USA). Results shown are representative of at least three experiments. Western densitometric measurements of the bands were quantified using the Labworks 4.6 Image Acquisition and Analysis software (UVP Ltd., Cambridge, UK). Briefly, the blot images were imported into the software and the area around each band was selected and the background intensity was subtracted from the blot images. The bands were then selected by drawing a tight boundary around them and the intensity of the bands was displayed in an excel format. To account for inhomogeneous protein loading the process was repeated for the western blot loading control and the ratio of the reference protein to the target protein was calculated. Finally all the values were expressed as a ratio relative to control.

**TGF-β ELISA:** The effect of FK506 on secreted TGF-β1 protein levels in HK-2 cell supernatants was assessed using a TGF-β ELISA (Cat # DB100B, R&D Systems, Minneapolis, MN, USA) The specificity and sensitivity of the assay was assessed using 5 ng of TGF-β1 as a positive control and sterile water as a negative control.

**Real-time PCR:** Following cell treatment total RNA was isolated and purified utilizing the RNeasy mini kit (QIAGEN, Valencia, CA, USA). 5 µg of RNA was used to synthesis cDNA using the Superscript III First-Strand Synthesis System (Invitrogen, Carlsbad, CA, USA) as per the manufacturer’s instructions. The TaqMan^®^ Gene Expression Assays and TaqMan Universal PCR Master Mix (Applied Biosystems, Foster, CA, USA) were used for the quantitative real-time PCR analyis of the genes of interest (TGF-β1 Hs00998133_m1; α-SMA Hs00178696_m1; fibronectin 1 Hs00159940_m1; MMP-9 Hs00234576_m1, E-cadherin Hs00170423_m1 and vimentin Hs00185584_m1) and detected by Applied Biosystems 7900HT Fast Real-Time PCR System (Applied Biosystems, Foster, CA, USA). Data was processed using the QPCR Delta Ct method of analysis and an endogenous control, 18S ribosomal RNA (rRNA), was employed to allow for normalization of the target genes. All real-time PCR results were expressed a percentage change in gene expression relative to time-matched controls. Data represented are from four independent experiments.

**Gelatin Zymography:** Cell supernatants were concentrated using Amicon Ultra-4 Centrifugal Filter Devices (Millipore, Billerica, MA, USA). Samples were loaded onto a 10% SDS-PAGE gel containing 1 mg/mL of gelatin (Bio-Rad Laboratories, Inc., Hercules, CA, USA). After electrophoresis, the separated proteinases were renatured within the gel by replacing the SDS by washing the gel for three 20 min washes in a non-ionic detergent, 2.5% (*v*/*v*) Triton × 100. The gels were then incubated for 20 h at 37 °C in an enzyme activation buffer (50 mM·Tris, 10 mM·CaCl_2_, 50 mM·NaCl, 0.05% (*v*/*v*) Brij 35, 1.65 mM·NaN_3_). The gels were then stained with 0.25% (*v*/*v*) Brilliant Blue Coomassie for 1 h, prior to destaining in a solution of 30% (*v*/*v*) methanol, 10% (*v*/*v*) glacial acetic acid. Proteinases with gelatinolytic activity were visualized as clear areas of lytic activity against a blue-stained background of undigested protein.

**Statistical Analysis:** Statistical analyses were performed using GraphPad Prism 4.0. Data was analyzed by one-way analysis of variance (ANOVA) and multiple comparisons between the control and treatment groups were made using the Dunnett post-test. Comparisons between different treatment groups were made by the Bonferroni post-test. Alternatively, where appropriate, confidence intervals were constructed (95%) and an unpaired Students t-test was used to test for statistical significance. Results were expressed as the mean ± standard error of the mean (SEM). A probability of 0.05 of less was deemed statistically significant.

## 3. Results and Discussion

### 3.1. FK506 Treatment Resulted in Significant Increases in Both LDH Release and Resazurin Conversion Without Affecting RPTEC Proliferation

**Figure 2:**
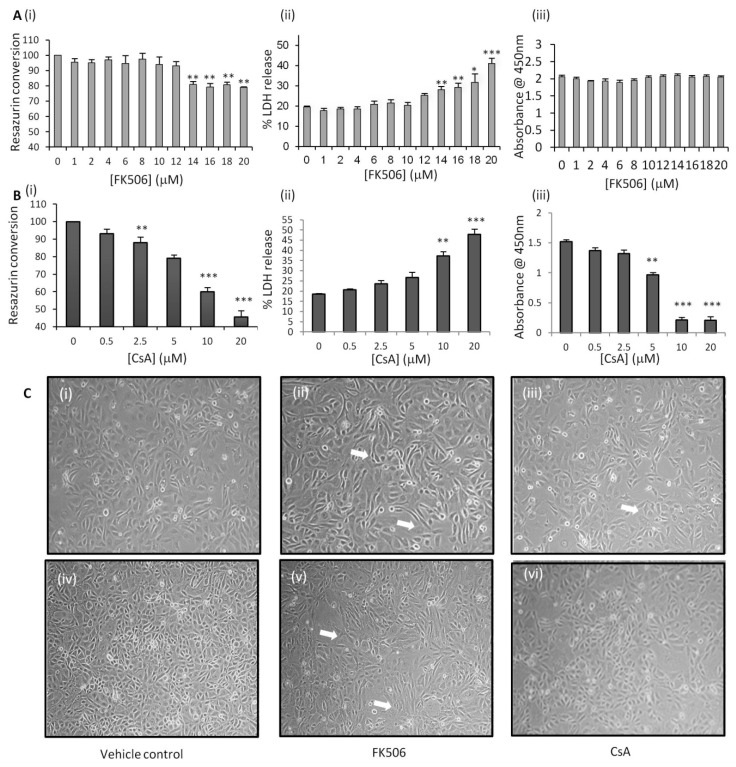
Effects of FK506 on HK-2 cells. HK-2 cells cultured on 96-well plates were treated with (**A**) FK506 across a concentration range of 0–20 μM or (**B**) CsA across a concentration range of 0–20 µM for 48 h. (i) Medium was aspirated and cells were incubated for 2 h at 37 °C in the presence of 0.1 mg/mL resazurin. Fluorescence was then read at excitation wavelength 530 nm and emission wavelength 590 nm. Results are expressed as percentage control at each time-point and represent the mean + SEM (*n* = 4), ** *p* < 0.01 *vs.* time-matched vehicle treated control cells; (ii) lactate dehydrogenase (LDH) activity was assayed in supernatant and whole cell samples using a specific LDH activity assay (Sigma). Absorbance was read at 590 nm and results are expressed as percentage LDH release at each time-point and represent the mean + SEM (*n* = 4): * *p* < 0.05, ** *p* < 0.01, *** *p* < 0.001 *vs.* time-matched vehicle treated control cells; (iii) HK-2 proliferation was assessed by quantification of BrdU incorporation using a specific BrdU assay (Calbiochem). Shown are absorbance readings @ 450 nm that represent the mean + SEM (*n* = 4); (**C**) HK-2 cells were cultured on 6-well plates and treated with vehicle control or medium containing 5 µM FK506 for 12 (i + ii + iii) or 48 h (iv + v + vi). Phase contrast micrographs were taken using a CCD camera mounted on a Nikon microscope (Magnification 10×). Arrows indicate changes in cell morphology. Images are representative of at least five independent experiments.

Initially, the dose dependent effects of FK506 (concentrations ranging from 0–20 µM) on HK-2 cells was examined. HK-2 cell viability at 48 h was assessed by the resazurin reduction assay and the results demonstrated that 12 μM FK506 resulted in no significant change in resazurin conversion when compared with control cells, while 14–20 µM FK506 resulted in statistically significant decreases in resazurin reduction (Figure 2A(i)), suggesting reduced HK-2 cell viability following FK506 treatment. In comparison 48 h exposure to the known nephrotoxic immunosuppressant CsA didn’t induce a significant reduction in resazurin at 0.5–2.5 µM CsA concentrations compared to control cells; however at concentrations in excess of 5 µM resulted in significantly decreased levels of resazurin reduction (Figure 2B(i)).

To further investigate the cytotoxic effects of FK506, the release of the cytosolic enzyme LDH from HK-2 cells following 48 h exposure to varying concentrations of FK506 was assessed (Figure 2A(ii)). A statistically significant increase in levels of LDH release was observed with FK506 concentrations of 14–20 μM, compared to control cells, indicating increased cellular damage. A similar trend was observed following CsA exposure, with a statistically significant increase in LDH detected compared to control following exposure to 10–20 µM CsA (Figure 2B (ii)).

The BrdU assay determined that FK506 has no effect on HK-2 cell proliferation at all tested concentrations (Figure 2A(iii)). CsA exhibited a dose-dependent effect on BrdU incorporation into HK-2 cells. CsA concentrations ranging from 0.5–2.5 µM exhibited no significant reduction in BrdU incorporation, however 48 h exposure to CsA concentrations ranging from 5–20 µM induced a statistically significant decrease in BrdU incorporation compared to control cells, indicating decreased HK-2 cell proliferation (Figure 2B(iii)).

Analysis of the cytomic data profiles of FK506 allowed the determination of a sub-cytotoxic dose for use in the experimental model. Based on the results of the cytomic assays and current knowledge relating to the efficacy of FK506 *in vivo*, a FK506 dose of 5 µM was chosen for further analysis of the differential effects of this immunosuppressant on renal proximal tubular epithelial cells. Similarly, in this HK-2 model, 5 µM·CsA was selected as the dosage for subsequent experiments.

### 3.2. FK506 Treatment Induced Morphological Alterations in the RPTECs

Given that alterations in cell morphology are reflective of major changes in the form and function of cells, the effect of 5 μM FK506 on HK-2 cell morphology was assessed using phase contrast microscopy (Figure 2C). HK-2 cells were treated at 90% confluency and phase contrast micrographs were taken at 12 and 48 h post-treatment. Control cells (Figure 2C(i, iv)) exhibited a typical cuboidal epithelial morphology with tight, regular cell-cell junctions and a high degree of attachment between neighboring cells. FK506-treated cells exhibited notable morphological differences compared to the control cells at both 12 and 48 h (Figure 2C(ii, v)), with an observable loss of the normal tight cell–cell adhesion and cells exhibiting a more elongated phenotype suggestive of a fibroblast-like phenotype. Treatment with the nephrotoxic immunosuppressant CsA exerted time dependent effects on cell morphology. CsA treated cells were observed to lose tight cell-cell adhesion over time and exhibit a more elongated phenotype suggestive of a fibroblast-like phenotype. Following 48 h exposure to CsA the treated cells exhibited widespread gaps in the monolayer with fewer cells visible (Figure 2C(iii, vi)).

### 3.3. FK506 Induced Myofibroblast Transition in the RPTEC Cells

To explore the ability of FK506 to initiate EMT processes within a renal epithelial cell line, HK-2 cells were cultured in the presence of 5 µM FK506 or 5 µM CsA for 12 and 48 h. The effects on the expression of known markers of myofibroblast activation, e.g., fibronectin, metallomatrix proteins (MMPs), vimentin, α-SMA, and markers of epithelial junctional integrity, e.g., E-cadherin, were examined.

**Figure 3:**
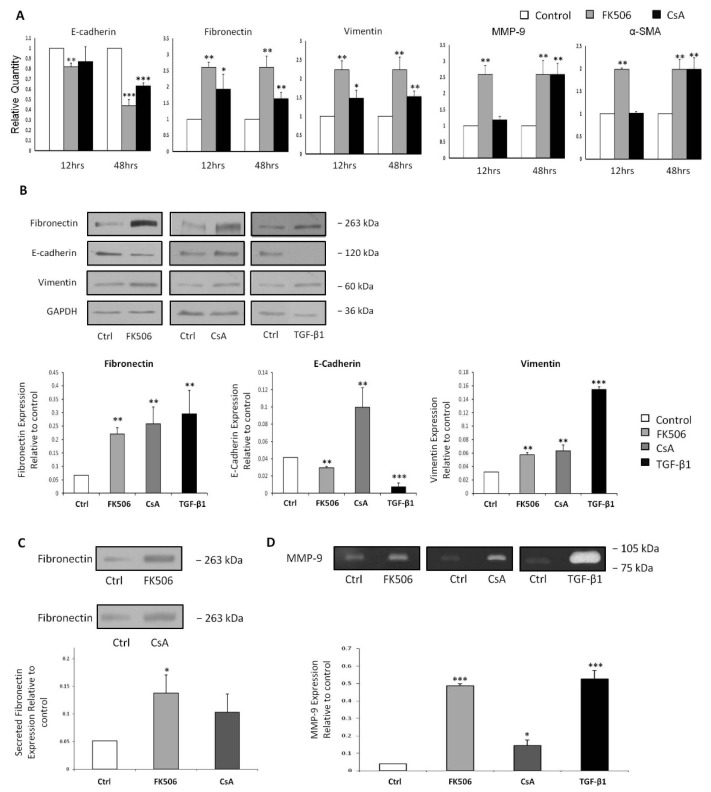
The effect of FK506 treatment on classical EMT markers. HK-2 RPTECs were cultured in 6-well plates and treated with control medium or medium containing 5 µM FK506 or 5 µM CsA for the indicated time periods. (**A**) RNA was isolated and cDNA synthesised. Real time RT-PCR was then carried out on the cDNA using primers specific to E-cadherin, fibronectin, vimentin, MMP-9 and α-SMA. 18S RNA was used as a total RNA control throughout. Data is expressed as a % change in mRNA levels relative to time-matched control ± SEM (*n* = 4): * *p* < 0.05, ** *p* < 0.01 *vs.* time-matched vehicle treated control cells; (**B**) HK-2 cells were cultured on 6-well plates and treated with 5 µM FK506, 5 μM CsA or 5 ng/mL TGF-β1 for 48 h and whole cell lysates were prepared. Equal amounts of protein were separated by SDS-PAGE electrophoresis, transferred to nitrocellulose and indirectly probed for fibronectin, E-cadherin and vimentin. GAPDH was used as a loading control. A representative blot is shown from three independent experiments; (**C**) HK-2 cells were cultured on 6-well plates and treated with 5 µM FK506 or 5 μM CsA for 48 h. Cell culture supernatants were harvested, centrifuged to remove cellular debris and concentrated using Amicon Ultra-4 Centrifugal Filter Devices (Millipore). Concentrated supernatants were separated by SDS-PAGE electrophoresis, transferred to nitrocellulose and indirectly probed for fibronectin using a mAb and ECL detection system. A representative blot is shown from three independent experiments; (**D**) HK-2 cells were cultured on 6-well plates and treated with 5 µM FK506, 5 μM CsA or 5 ng/mL TGF-β1 for 48 h. Cell culture supernatants were harvested, centrifuged to remove cellular debris and concentrated using Amicon Ultra-4 Centrifugal Filter Devices (Millipore). Gelatin substrate zymography was used to separate and assess MMP-9 activity. Clear areas represent areas of gelatinolytic activity. A representative zymogram is shown from three independent experiments.

As a marker of EMT the effects of FK506 treatment on fibronectin induction in RPTEC cells was examined. Fibronectin mRNA levels were increased significantly relative to control (** *p* < 0.01) following 5 μM FK506 or 5 µM CsA treatment at both 12 h and 48 h (Figure 3A). These elevations in fibronectin mRNA levels correlated with the increases seen at whole cell protein levels following 48 h treatment with either 5 µM FK506 or 5 µM CsA (Figure 3B). Exposure to 5 ng/mL TGF-β1 was employed as a positive control for the initiation of EMT, resulting in a significant increase in vimentin protein expression in comparison to the time-matched controls (*p* < 0.01) (Figure 3B). The secretion of globular, soluble fibronectin is an essential step in the cell-mediated conversion of fibronectin to its fibrillar form, and its incorporation into the connective tissue environment. To investigate whether the observed immunosuppressant effects on the secreted fibronectin levels reflected the transcriptional and whole cell protein levels, fibronectin concentrations in supernatants from immunosuppressant treated RPTEC cells were assessed by Western blot analysis. Treatment with 5 µM CsA resulted in elevated fibronectin secretion, although this increase failed to reach statistically significant levels. Conversely, exposure to 5 μM FK506 resulted in significantly elevated levels of fibronectin in concentrated supernatants at 48 h compared to the time-matched controls (Figure 3C).

The effect of immunosuppressant treatment on MMP-9 transcription was investigated as it has been demonstrated to have a role in the degradation of the epithelial basement membrane. Treatment with 5 μM FK506 resulted in significant increases in MMP-9 mRNA levels at both the 12 and 48 h time-point (Figure 3A) (** *p* < 0.01). Treatment with 5 µM CsA resulted in a significant increase in MMP-9 mRNA at 48 h (Figure 3A) (*p* < 0.01) MMP-9 is secreted into the pericellular space as an inactive pro-enzyme, requiring post-translational modification to induce its ECM proteolytic activity. In order to investigate the functional activity of MMP-9, reflecting its *in situ* degradative potential, concentrated RPTEC cell supernatants harvested after 48 h exposure to either 5 µM FK506, 5 µM CsA or 5 ng/mL TGF-β1 were assessed by gelatin zymography. TGF-β1 was employed as a positive control for the gelatin zymography as it is expected that TGF-β1 exposure should induce significantly increased MMP-9 activity [31] (*p* < 0.001). Both CsA and FK506 induced statistically significant increases in MMP-9 activity as evidenced by the gelatin zymography (*p* < 0.05 and *p* < 0.001 respectively) (Figure 3D), correlating well with the previously observed increases in MMP-9 mRNA (Figure 3A).

Vimentin is the most abundant intermediate filament protein in various cell types, including smooth muscle cells [32] and its up-regulation is widely accepted as a myofibroblast marker. Vimentin mRNA levels were increased significantly (** *p* < 0.01) following both 5 μM FK506 and 5 µM CsA treatment at both 12 and 48 h time points when compared to respective time-matched controls (Figure 3A). The observed increases in vimentin mRNA levels correlate with increased fibronectin protein expression observed 48 h (Figure 3B). CsA resulted in significant increases in vimentin mRNA expression in comparison to time matched controls at both 12 and 48 h (*p* < 0.05 and *p* < 0.01 respectively) (Figure 3A). These findings were mirrored by a significant increase in vimentin protein expression at 48 h (*p* < 0.01) (Figure 3B). As an accepted initiator of EMT, it was found that TGF-β1 induced a significant increase in vimentin protein levels (*p* < 0.001) (Figure 3B).

α-SMA is an actin isoform characteristic of smooth muscle cells [33,34] and in an EMT setting indicates full differentiation into an activated myofibroblast phenotype. In our model, FK506 treatment induced a significant (** *p* < 0.01) increase in mRNA expression of α-SMA at both 12 and 48 h relative to time-matched controls (Figure 3A). Exposure to CsA also induced a significant increase in α-SMA expression at 48 h compared to time-matched controls (*p* < 0.01) (Figure 3A).

The integrity of the epithelial adherens-junctions was assessed by examining the expression of E-cadherin, a critical component of the epithelial adherens-junction. E-cadherin mRNA levels were significantly reduced following 5 μM FK506 treatment at both 12 h (*p* < 0.01) and 48 h time-point (*p* < 0.001) when compared to time-matched controls. This reduction in E-cadherin mRNA levels correlated with the decreases seen at whole cell protein levels at 48 h (*p* < 0.01) (Figure 3B). E-cadherin mRNA levels were not significantly affected by CsA exposure for 12 h compared to control, however 48 h exposure to CsA resulted in a significant reduction in E-cadherin mRNA levels compared to time-matched controls (*p* < 0.01) (Figure 3A). Subsequent examination of the whole cell E-cadherin protein levels by western blot showed no correlation with the decreased mRNA levels. In contrast to the mRNA expression, treatment with CsA resulted in a significant increase in E-cadherin expression in HK-2 cells (Figure 3B). Exposure to TGF-β1 for 48 h resulted in significant loss of E-cadherin protein expression compared to time-matched controls (*p* < 0.001) (Figure 3B).

### 3.4. FK506 Increased TGF-β1 mRNA Levels and TGF-β1 Peptide Release from RPTEC Cells, with Concomitant Increases in Phospho-Smad2 Whole Cell Protein Levels

**Figure 4:**
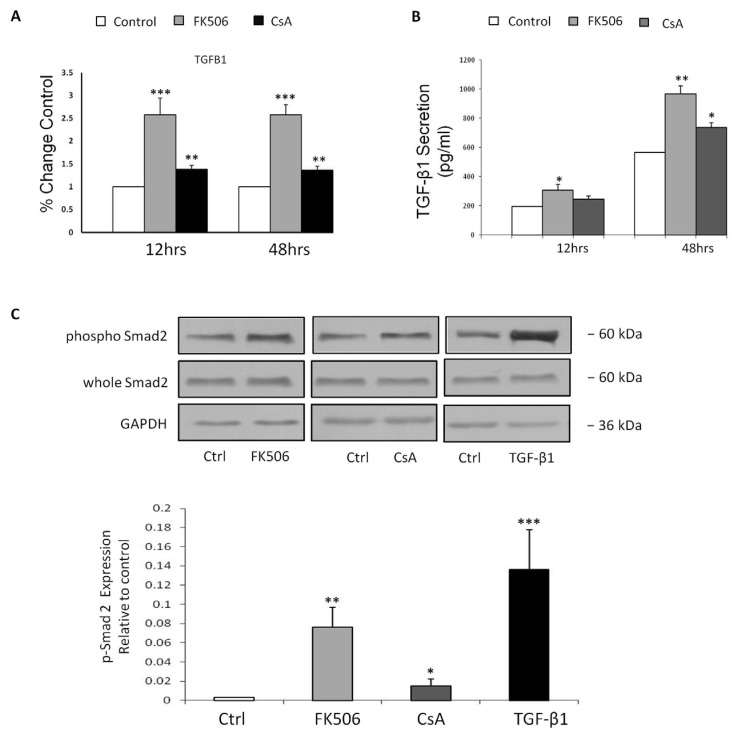
Effect of FK506 treatment on HK-2 TGFβ-1 mRNA levels, secretion levels and whole cell levels of phosphorylated Smad-2. HK-2 cells were cultured in 6-well plates and treated with control medium or medium containing 5 µM FK506 or 5 µM CsA for the indicated time periods. (**A**) RNA was isolated and cDNA synthesised. Real time RT-PCR was then carried out on the cDNA using primers specific to TGF-β1. 18S RNA was used as a total RNA control throughout. Data is expressed as a relative change in mRNA levels relative to time-matched control ± SEM (*n* = 4): * *p* < 0.05, ** *p* < 0.01 *vs.* time-matched vehicle treated control cells; (**B**) Cell supernatants were harvested at the indicated time-points and TGF-β1 levels were detected using a TGF-β1 ELISA (R&D Systems). Data is expressed as pg/mL TGF-β1 and represents the mean concentration ± SEM (*n* = 4): * *p* < 0.05, ** *p* < 0.01 *vs.* time-matched vehicle treated control cells; (**C**) Whole cell protein was extracted at 48 h with RIPA buffer. Equal amounts of protein were separated by SDS-PAGE electrophoresis, transferred to nitrocellulose and indirectly probed for phospho-Smad2. Whole cell Smad2 was used as a loading control. A representative blot is shown from three independent experiments.

TGF-β1, a multi-functional cytokine with fibrogenic properties, has been implicated in the pathogenesis of renal fibrosis and has been observed as a potential mediator of EMT in various *in vitro* cell culture models [35,36]. The effect of FK506 treatment on TGF-β1 induction in HK-2 cells was assessed as a potential mediator of EMT in our model. TGF-β1 mRNA levels were assessed by real-time RT-PCR at 12 and 48 h (Figure 4A). Following 5 µM FK506 treatment, TGF-β1 mRNA levels were increased significantly compared to time-matched controls at 12 h (* *p* < 0.05) and the 48 h time-point (*** *p* < 0.001) (Figure 4A). CsA exposure resulted in significantly elevated TGF-β1 mRNA levels at both 12 and 48 h (*p* < 0.01) (Figure 4A). To investigate whether transcriptional changes observed reflected levels of TGF-β1 release from RPTEC cells, TGF-β1 levels in supernatants were assessed using a TGF-β1 ELISA. 5 µM FK506 treatment resulted in a statistically significant increase in levels of TGF-β1 secretion at both 12 h (* *p* < 0.05) and 48 h (** *p* < 0.01) as compared to respective time-matched controls (Figure 4B). Exposure to 5 µM CsA resulted in a significant increase in TGF-β1 secretion at 48 h (*p* < 0.05) (Figure 4B).

Although TGF-β1 can elicit signaling responses through other pathways, Smad proteins are the specific intracellular effector molecules activated by TGF-β1. In order to investigate possible activation of the TGF-β/Smad axis by the immunosuppressants FK506 and CsA, whole cell protein levels of phosphorylated Smad2 were investigated by Western blotting, as an indicative marker of the TGF-β1-triggered Smad signaling cascade. Exposure to 5 ng/mL TGF-β1 was employed as a positive control for activation of the TGF-β/Smad axis. All treatments resulted in significant increases in phosphorylated Smad protein levels, with FK506 inducing a greater increase in expression than CsA compared to time-matched controls (*p* < 0.01 and *p* < 0.05 respectively) (Figure 4C). The increased levels of TGF-β1 peptide release from the HK-2 RPTEC cells correlated with the observed increase in whole cell protein levels of phosphorylated Smad2 (Figure 4C).

### 3.5. Treatment of RPTEC cells with FK506 Induced Increased Cell Migration Which Was Alleviated Following Co-Treatment with a TGF-β RI Kinase Inhibitor

**Figure 5:**
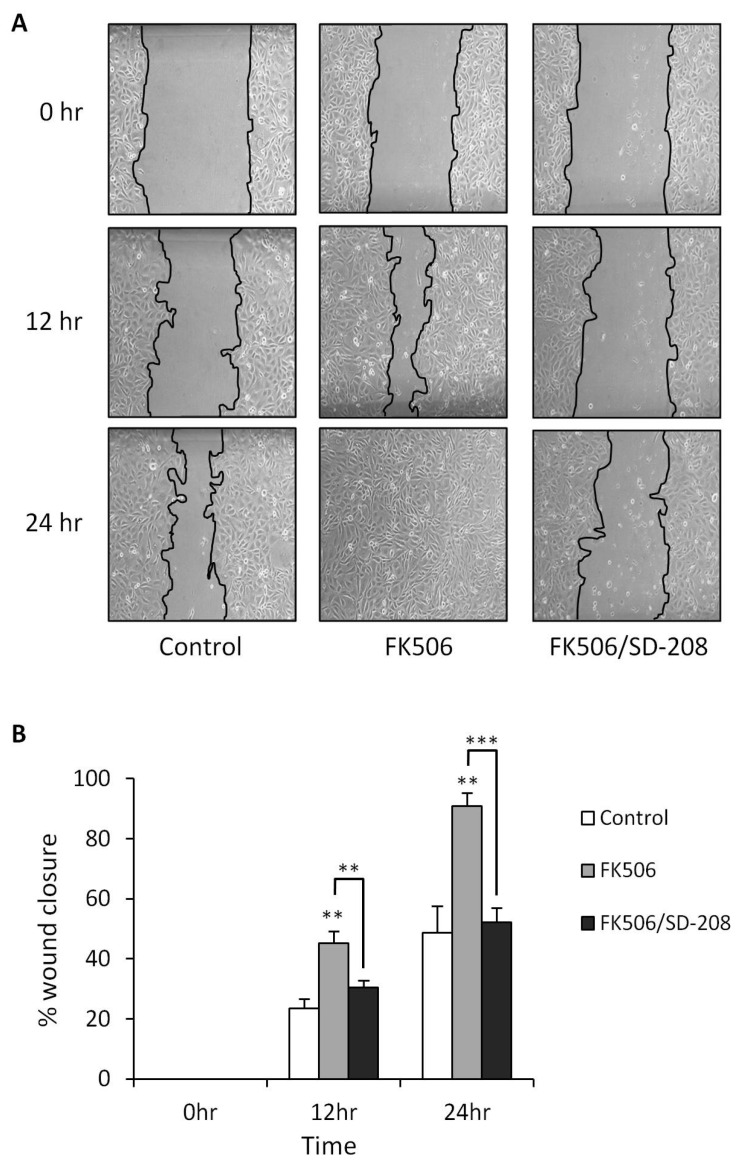
Treatment of HK-2 RPTECs with FK506 induced increased cell migration which was alleviated following co-treatment with a TGF-β RI kinase inhibitor (SD-208). (**A**) HK-2 RPTECs were cultured in 12 well plates. At the time of assay, wounds were inflicted on the cell mono-layer using a 200 μL pipette tip. The mono-layer was then washed twice with PBS and then treated with control medium, medium containing 5 μM FK506 alone or a co-treatment of FK506 and SD-208. Phase contrast images were then taken at 0, 12 and 24 h time-points. Representative images are shown from four independent experiments; (**B**) Images were analyzed using ImageJ software to calculate wound area. Data is expressed as the mean values of percentage wound closure relative to the corresponding 0 h time point and represent the mean percentage closure ± SEM (*n* = 4): *** *p* < 0.001; ** *p* < 0.01 *vs* time-matched vehicle treated control for each time-point.

An enhancement in cell motility is a fundamental and basic requirement of the EMT process. Consequently, due to the importance of an increase in a migratory phenotype, RPTEC motility was assessed using the scratch assay by visually assessing the ability of the cells to migrate into a denuded area. This was quantitatively assayed by measuring the area of the scratch at the same point, at time-points 0, 12 and 24 h using ImageJ software (Figure 5A). Values were then expressed as percentage of the total wound area covered by migrating cells. 5 μM FK506 treatment resulted in a statistically significant increase (** *p* < 0.01) in cell migration compared to vehicle treated control cells after 12 and 24 h (12 h 45% ± 4% wound closure *versus* 23% ± 3% closure; 24 h 91% ± 4% wound closure *versus* 48% ± 9%) (Figure 5B). Co-treatment of RPTEC cells with 5μM FK506 and 500 nM of a TGF-β RI kinase inhibitor (SD208) significantly abrogated FK506-induced migration at 12 h (45% ± 4% wound closure as opposed to 30 ± 2% closure; ** *p* < 0.01) and 24 h (91% ± 4% wound closure as opposed to 52% ± 5% closure; *** *p* < 0.001).

### 3.6. Pre-Incubation with a TGF-β Receptor 1 Kinase Inhibitor Completely Blocks Smad 2 Activation and Prevents Induction of EMT Markers by FK506

**Figure 6:**
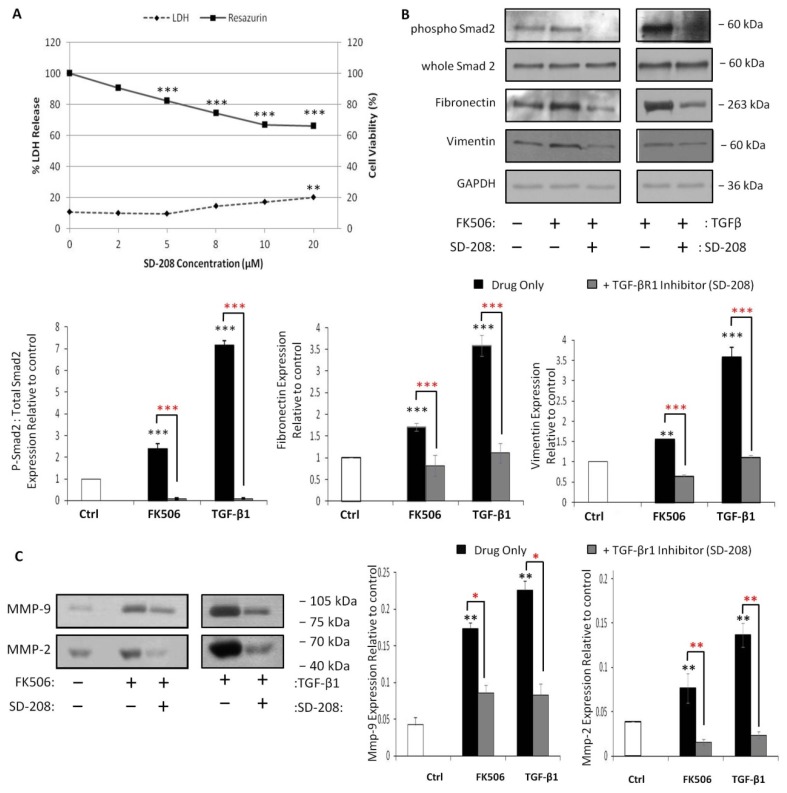
The specific blockade of TβRI kinase ablated Smad2 activation and dramatically affected the induction of the EMT markers, vimentin and MMPs by FK506. HK-2 cells were cultured on 6-well plates and treated with vehicle control, 5 µM FK506 with or without 500 nM of a TGFβRI/ALK5 kinase inhibitor V (SD-208). (**A**) Cells were incubated for 48 h with SD-208 concentrations ranging from 0–20μM. LDH and resazurin assays were conducted to assess SD-208 inhibitor toxicity; (**B**) Whole cell protein was extracted at 48 h with RIPA buffer. Equal amounts of protein were separated by SDS-PAGE electrophoresis, transferred to nitrocellulose and indirectly probed for vimentin and fibronectin using a mAb and ECL detection system. Whole Smad2 and GAPDH was used as a loading control. A representative blot is shown from three independent experiments. Densitometry is shown for the replicate blots; (**C**) Cell culture supernatants were harvested, centrifuged to remove cellular debris and concentrated using Amicon Ultra-4 Centrifugal Filter Devices (Millipore). Gelatin substrate zymography was used to separate and assess MMP-9 and MMP-2 activity. Clear areas represent areas of gelatinolytic activity. A representative zymogram is shown from three independent experiments. ** and *** indicates different levels of significant compared to time-matched control; *, ** and *** indicates varying levels of significance compared to treatment + inhibitor.

SD-208 (TGF-β1RI/ALK5 kinase inhibitor V) acts as a potent and selective inhibitor of TGF-β1RI/ALK5 kinase, thereby inhibiting TGF-β signaling through the prevention of Smad protein activation. Initially, the dose dependent effects of SD-208 (concentrations ranging from 0–20 µM) on HK-2 cells was examined. HK-2 cell viability at 48 h was assessed by the resazurin reduction assay and the results demonstrated that 0–2 μM SD-208 resulted in no significant change in resazurin conversion when compared with control cells, while 5–20 µM SD-208 resulted in statistically significant decreases in resazurin reduction (Figure 6A), suggesting reduced HK-2 cell viability. To further investigate the cytotoxic effects of SD-208, the release of the cytosolic enzyme LDH from HK-2 cells following 48 h exposure to varying concentrations of SD-208 was assessed (Figure 6A). A statistically significant increase in levels of LDH release was observed only at concentrations of 20 µM SD-208, compared to control cells, indicating increased cellular damage (Figure 6A). A concentration of 500 nM was chosen for future experiments due to the lack of toxicity in the HK-2 cell line.

To investigate the potential relevance of the TGF-β/Smad signaling pathway in the observed response to FK506 treatments the effects of exposure on Smad signaling and the expression of mesenchymal markers was assessed in combination with SD-208, a TGFβ1R1/ALK5 kinase inhibitor V, which inhibits TGF-β signaling through the prevention of Smad activation. Treatment of HK-2 cells with 5 µM FK506 or 5 ng/mL TGF-β1 resulted in a significant increase in the activation levels of Smad2 at 48 h (*p* < 0.001) (Figure 6B). Pre-treatment of HK-2 cells with SD-208 (500 nM) significantly abrogated levels of Smad2 phosphorylation following 48 h incubation with 5 µM FK506 or 5 ng/mL TGF-β1 (*p* < 0.001) (Figure 6B). 

The involvement of the TGF-β/Smad signaling pathway in the expression of mesenchymal markers induced by FK506 and TGF-β1 was assessed in the presence and absence of SD-208. 5 µM FK506 was shown to result in significantly increased levels of fibronectin and vimentin (*p* < 0.001 and *p* < 0.01 respectively) (Figure 6B). TGF-β1 also resulted in significantly increased cellular levels of fibronectin and vimentin (*p* < 0.001) (Figure 6B). Pre-treatment with SD-208 significantly decreased the FK506 and TGF-β1-induced increases in these mesenchymal markers (*p* < 0.001) (Figure 6B).

To investigate the involvement of the TGF-β/Smad signaling pathway in the alterations in MMP activity levels induced by 5 µM FK506 at 48 h, cells were treated with the normal treatment regimen in the presence and absence of SD-208 (500 nM). Pre-treatment with SD-208 significantly abrogated the FK506-induced increase in MMP-9 and MMP-2 activity levels (Figure 6C). This finding is supported by a similar finding following TGF-β1 exposure (Figure 6C). This suggests that the activation of the TGF-β/Smad pathway is necessary for the elaboration of the EMT response induced by FK506.

## 4. Conclusions

In order to gain an insight into the molecular mechanisms involved in FK506-induced renal injury, an appropriate *in vitro* experimental model system, the human renal proximal tubular epithelial cell line, HK-2 RPTEC cells, was selected. The selection of the RPTEC cell line was based on the fact that this cell line has been utilized extensively in the investigation of CsA nephrotoxicity (also a calcineurin inhibitor) and because the epithelial cells of the proximal tubule are the target sites for various nephrotoxic agents *in vivo* [37,38].

The determination of the most relevant drug concentration to employ is central to the successful application of any *in vitro* model in the assessment of nephrotoxicity. Problems arise in this respect based on the capability of the intact kidney *in vivo* to concentrate compounds during urine formation to a degree that may exceed plasma concentrations by a factor of 10^2^ to 10^3^ [39]. Therefore the classical approach to renal cell culture is not readily applicable to “chronic” exposures. Studies involving the application of the test compound once to the culture medium can be considered analogous to an acute exposure. The employment of high concentrations of nephrotoxins *in vitro* may be required to mimic long-term chronic exposure *in vivo*. Indeed, many studies have employed high acute doses of the drug as a predictive indicator for low dose chronic exposure [40].

For the purposes of this study, sub-cytotoxic doses of FK506 and CsA were determined. The cytomic assays identified that a concentration of 14 µM FK506 resulted in a significant increase in both LDH release and resazurin conversion. With this in mind, the sub-cytotoxic dose concentration for FK506 is 12 µM. However, based on the fact that FK506 is more potent than the other commonly used immunosuppressant, CsA [41], with dosage levels exceeding 100 times those of CsA, a concentration of 12 µM would be unrealistic in the *in vivo* setting, even when taking the kidney’s ability to concentrate toxins into account. Therefore, a concentration of 5 µM FK506 was selected for use in this study due to the fact that in the *in vitro* scenario, high single doses of drugs are deemed necessary to mimic long-term chronic exposure *in vivo*. Similarly, in this HK-2 model, 24 h exposure to 5 µM CsA resulted in a significant decline in resazurin conversion without a concomitant significant increase in LDH.

Cellular senescence refers to the state in which normal cells stop dividing and enter an irreversible state of growth arrest while remaining viable and metabolically active. The capacity for tissue renewal and repair is accepted to deteriorate with time. The numbers of senescent cells have been shown to increase with age in renewable tissues, and are also present at sites of chronic age-related pathology such as atherosclerosis [42,43,44]. Hence, senescence may contribute to a gradual reduction in tissue renewal and function, which is an important consideration in terms of the kidney especially after nephrotoxic exposure. In this model 48 h exposure to CsA resulted in diminished proliferation and DNA synthesis, however care must be taken in classifying these results as part of the induction of a stress-induced senescent phenotype. Proliferation capable cells (especially tubular epithelial cells) can spend long intervals in a reversibly arrested quiescent state, and in response to appropriate signals including the need for tissue repair and regeneration proliferation is resumed. Initial results with CsA in our model, in combination with other studies in the literature, adds credence to the concept that CsA may exert some of its deleterious effects via the induction of a stress-induced senescent phenotype. Long-term CsA-induced increases in senescent cell numbers among PTECs *in vivo* would lead to important complications in transplant recipients. Nephrotoxicity induced cell damage can lead to elimination of the affected cells by apoptosis, however, neighboring senescent cells, which lack the ability to proliferate and replace damaged epithelium, may exacerbate resulting tubular atrophy. Conversely, even with 20 μM treatment for 48 h, FK506 failed to effect HK-2 cell proliferation compared with 5 μM CsA exposure, regardless of its superior potency. The effect of FK506 on proliferation may be cell-type specific, as its beneficial effects in treatment of autoimmune glomerulonephritis has been credited to an anti-proliferative action on excessive mesangial cell expansion [45].

EMT, which plays a role in governing embryonic morphogenesis in multicellular organisms, can be reactivated in adults during fundamental processes such as wound healing and tissue regeneration but also pathologically in fibrosis and cancer. Various studies have highlighted the potential contribution of EMT in the development of TIF, particularly highlighting CsA-induced EMT as a key event in the development of interstitial fibrosis [46,47]. Cellular morphology is a basic indicator of cellular phenotype. Proximal tubular epithelial cells are polarized and cuboidal in shape, with highly developed intercellular junctional structures. FK506 induces phenotypic changes, with RPTEC cells becoming elongated in shape, dissociating from neighboring cells and losing their cobblestone monolayer pattern.

One of the central findings in this study was that FK506 treatment was demonstrated to increase the migratory ability of RPTEC cells as determined by scratch wound assay. In terms of the fibrotic renal setting, the ability of FK506 to increase the migration of EMT-derived myofibroblasts into the tubulointerstitium could significantly contribute to the development of TIF due to the fact that once re-located in the tubulointerstitium, myofibroblasts can increase ECM deposition and release pro-fibrotic factors that may induce other TECs to undergo EMT, thereby initiating a cycle of fibrosis. Additionally, increased migration of EMT-derived myofibroblasts may further contribute to a decline in renal function by disrupting tubular epithelium homeostasis, thereby facilitating tubular atrophy. In our model, FK506’s ability to increase RPTEC cell migration was coupled with increased fibronectin, vimentin, α-SMA and MMPs expression with concomitant down-regulation of E-cadherin expression.

Increased ECM deposition is a central aspect of fibroblast biology, providing a temporary scaffold for the granulation tissue, which facilitates re-epithelialisation of the wound area. However, in chronic fibrotic disorders inappropriate and unchecked deposition of fibronectin occurs and fibronectin functions as a mediator of interstitial fibrotic expansion and renal architectural deterioration [48]. In our model, FK506 treatment induced increased fibronectin expression at both the mRNA and protein level in RPTEC cells. FK506 augmentation of fibronectin expression has previously been demonstrated in both a rat model of chronic FK506 nephrotoxicity [49] and in human renal biopsies from patients with histologically confirmed FK506 nephrotoxicity [50]. During EMT, fibronectin can have dual functions, acting as a marker of ECM production as well as facilitating cell migration [51]. In terms of the EMT process, increased fibronectin production induced by FK506 not only contributes to progressive interstitial thickening but may also facilitate the migration of epithelial-derived myofibroblasts into the interstitium.

From an EMT perspective, vimentin and α-SMA expression are directly linked to an increased contractile capability [52,53]. Increased expression of α-SMA, an actin isoform characteristic of smooth muscle cells [33], is indicative of the complete differentiation of an epithelial cell into an activated myofibroblast, with this *de novo* expression enabling acquisition of enhanced motility and contractibility [54]. Previous studies have demonstrated the ability of FK506 to induce significant increases in α-SMA in both a rat renal isograft model and a streptozotocin-induced diabetic rat model [55,56] and in protocol renal transplant biopsies [57]. In our model, FK506 exposure induced a significant increase in mRNA expression of α-SMA at 48 h, supporting the suggestion of the acquisition of a myofibroblast phenotype. CsA exposure failed to induce a significant increase in a-SMA gene expression was detected at the earlier 12 h time-point, suggesting that α-SMA induction is a late stage event in this HK-2 model, a conclusion which is supported by previous publications which demonstrate α-SMA induction was not observed in TGF-β1 treated PTECs until 36–48 h [58]. A delayed significant induction of α-SMA was observed in this study following 48 h CsA exposure, suggesting a more phenotypic phenotype. This late induction of α-SMA suggests that TECs are reluctant to undergo phenotypic conversion under normal conditions, unless there is a sustained interstitial injury. Vimentin is the most abundant intermediate filament protein in various cell types, including smooth muscle cells [32] FK506 has previously been shown to increase vimentin protein expression in culture astrocytes derived from rats [59]. Polymerized vimentin filament networks enable the cell to cope with the application of dynamic stress encountered in actions such as cell spreading and motility [60]. It has been proposed that vimentin plays a role in mediating cell migration, as a result of increased cell motility capacity [60]. It has been proposed that vimentin plays a role in mediating cell ultra-structural stability under the application of cellular stress, such as that undertaken during migration [52]. In our model, both FK506 and CsA treatments induced a significant, time dependent increase in both mRNA and protein expression of vimentin, suggesting the acquisition of a more myofibroblast-like phenotype. TGF-β1 treatment was utilized as a positive control, inducing a statistically significant increase in vimentin protein expression.

In the normal kidney, tubular epithelial cells and interstitial fibroblasts are located in different biological compartments, separated by the tubular basement membrane (TBM). The degradation of the basement membrane encasing the basolateral aspect of the proximal tubular cells by the zinc-dependent endopeptidases, MMP-2 and MMP-9, has been identified as an important event in EMT progression as it facilitates migration from the tubule environs into the interstitial space [58]. Previous work by Lan reported an increase in MMP-9 activity in the supernatants of FK506 treated keratinocytes [61]. In our model system, FK506 induced increased MMP-9 and MMP-2 expression in RPTEC cells. Given their role in matrix degradation [62], the induction of MMPs as a pro-fibrotic mechanism seems counterintuitive; however, their role in global disease progression seems to be temporally dependent. In a fibrotic setting involving EMT, early induction of MMPs may be pathological as it facilitates EMT development. However, later induction of MMPs may be beneficial as it is thought to be an attempt at matrix degradation and inflammatory resolution. 

Given the physiological role of PTECs *in vivo* as a permeability barrier, it is not surprising that they are characterized by an extensive network of cell-cell junctions, which function to secure them into a tight epithelial cell monolayer creating an effective barrier to the luminal filtrate [63,64]. Cell junctional disruption has been repeatedly used as a marker of EMT both in cell culture and animal models of renal disease, and is thought to facilitate the acquisition of a more motile phenotype. One of the major epithelial cell–cell junctional assemblies is the adherens junction. E-cadherin is a prototypic member of the family and a principal component of adherens junctions in polarized epithelial cells [65]. Loss of E-cadherin is considered a hallmark of EMT, indicative of a disruption in adherens junctional integrity. In our model, treatment with FK506 induced a transcriptional and translational decrease in E-cadherin expression in RPTEC cells adding further credence to the hypothesis that FK506 is capable of inducing a myofibroblast transition in PTECs. CsA decreased E-cadherin mRNA expression, however, interestingly; this transcriptional reduction did not correlate with whole cell E-cadherin protein levels, where increased E-cadherin protein levels were observed. Although whole cell protein levels allow the global regulation of E-cadherin expression to be assessed, E-cadherin’s physiological function is governed by its incorporation into matured F-actin tethered adherens junction complexes. CsA has been demonstrated to induce endoplasmic reticulum stress in primary cultured TECs [66], with studies demonstrating that treatment of epithelial cells with endoplasmic reticulum stress inducers elevated o-glycolysaltion of newly synthesized E-cadherin protein in the cytoplasm and prevented its cell surface transport and incorporation into adhesion sites [67]. Junctional and polarity proteins are in a state of dynamic flux during cell migration [68]; however, it has recently been suggested that even in confluent epithelial cell monolayers these proteins may also be in a dynamic equilibrium between cytoplasmic and membrane-associated pools [69]. In this model it can be hypothesized that CsA may affect localization of E-cadherin to the cell junctions and possibly its degradation, therefore increasing overall E-cadherin cell levels.

The signaling mechanisms that are activated in response to immunosuppressive induced renal injury leading to TIF are not fully understood. Upon exposure of PTECs to immunosuppressive agents, the resulting epithelial micro injuries can potentially initiate a variety of cell type dependent signaling cascades and activity profiles, including epithelial apoptosis and EMT, which trigger fibrogenic loci and progressive fibrogenesis in chronic renal injury. EMT involves extensive alterations in gene expression and cytoskeletal re-organization in order to instigate a phenotype conversion. Logically, significant alterations in intracellular signaling cascades occur in order to facilitate large scale transcriptional re-programming. Numerous studies, in both transgenic mice models of TGF-β1 induced renal fibrosis [70] and human renal allograft biopsies [71], have identified a central role for TGF-β1 and its downstream signaling effectors in stimulating cellular mechanisms that promote the progression of renal disease [72]. The phenotype associated with chronic, progressive renal disease of interstitial scarring, tubular cell atrophy, loss of differentiated structures and nephron loss is consistently associated with up-regulation of TGF-β [73,74]. Critically, activation of TGF-β signaling has been shown to be sufficient to induce EMT in *in vitro* epithelial cell models [75,76] and in terms of our model system, TGF-β1 is widely considered a lead candidate for the mechanism of CsA-induced nephrotoxicity [37,50].

In our model, FK506 treatment of RPTEC cells resulted in elevated TGF-β1 mRNA levels which correlated with increased levels of TGF-β1 protein release, in agreement with published studies which demonstrated increased TGF-β1 gene expression in biopsies obtained from transplant patients with FK506 nephrotoxicity [50] and increased TGF-β1 protein staining in kidney sections derived from FK506-treated Sprague-Dawley rats [77]. Upon ligand binding by TGF-β1, type II TGF-β receptors (TβRII) activate type I TGF-β receptors (TβRI), recruiting and activating the intracellular mediators Smad2 and Smad3, which subsequently complex with Smad4 before translocating to the nucleus where the complex regulate gene transcription [78]. In order to examine whether significant increases in TGF-β1 secretion from RPTEC cells, induced by FK506 treatment, resulted in activation of the TGF-β/Smad signaling pathway, whole cell levels of phosphorylated Smad2 were investigated as an indicative marker of TGF-β-triggered Smad signaling cascade. FK506 treatment significantly increased Smad2 activations levels. As well as increasing extracellular TGF-β1 release, FK506 can potentially facilitate TGF-β/Smad signaling through its ability to interact with the cytoplasmic immunophilin FK506-binding protein 12 (FKBP12). FK506 interacts with FKBP12 in order to trigger immunosuppression; however, FKBP12 negatively affects TβRI signaling by binding to the receptor preventing phosphorylation, thereby blocking access to activators [79]. Through this interaction, it is theorized that FKBP12 protects against ligand independent activation of TβRI. In relation to our study, the FKBP12 ligand FK506 was observed to provoke the release of FKBP12 from the cytoplasmic tail of TβRI, causing kinase activation and Smad2 phosphorylation [80].

The activation of the TGF-β/Smad pathway is necessary for the amplification of the EMT response in RPTEC cells following FK506 treatment, since pre-incubation of RPTEC cells with a TGF-β receptor I kinase inhibitor completely blocked Smad2 activation in response to FK506 which dramatically affected the induction of the EMT markers, fibronectin, vimentin and MMP-9 and -2. Inhibition of TβRI function using a chemical inhibitor has been shown to block EMT and promote an enhanced epithelial phenotype in various cell culture studies [81,82,83,84]. FK506’s ability to increase the migratory capacity of RPTEC cells in our model was alleviated by specific blockade of TβRI kinase. This reduced migratory capacity may also be linked to the concomitant attenuation of FK506-induced fibronectin, vimentin and MMP expression owing to their possible requirement for an increased migratory capacity of epithelial-derived myofibroblasts.

In conclusion, the results presented in this study support the hypothesis that the calcineurin inhibitor FK506 is capable of inducing a myofibroblast transition in the HK-2 RPTEC cell line and that the activation of the TGF-β/Smad pathway was necessary for the induction of the EMT response. Consequently, this work supports the theory that EMT is a major mechanism in the development of renal fibrosis associated with CNI nephrotoxicity. 

## Abbreviations

α-SMA	Alpha smooth muscle actin
ANOVA	One way Analysis of Variance
BrdU	Bromodeoxyuridine
CaCl_2_	Calcium Chloride
CaN	Calcineurin Phosphatase
cDNA	Complimentary deoxyribonucleic acid
CNI(s)	Calcineurin inhibitor(s)
CKD	Chronic Kidney Disease
CO_2_	Carbon Dioxide
CpN	Cyclophilin
CsA	Cyclosporine A
Ctrl	Control
DMEM	Dulbecco’s modified Eagle’s medium
DMEM/F-12	DMEM/Nutrient mix F-12
DMSO	Dimethylsulfoxide
DNA	Deoxyribonucleic acid
ECM	Extracellular matrix
ELISA	Enzyme linked immunosorbent assay
EMT	Epithelial-Mesenchymal Transition
ESRD	End Stage Renal Disease
FK506	FK506, Tacrolimus
FKBP12	FK506 Binding Protein 12
HK-2	Human Kidney-2
kDA	Kilodaltons
LDH	Lactate Dehydrogenase
Mg	Milligrams
mM	Millimolar
MMP	Matrix metalloproteinase
NaCl	Sodium Chloride
NFAT	Nuclear Factor of Activated T-cells
NFAT_C_	Cytosolic Nuclear Factor of Activated T-Cells
NFAT_N_	Nuclear Nuclear Factor of Activated T-Cells
Ng	Nanograms
nM	Nanomolar
PCR	Polymerase chain reaction
Pg	Picograms
PTECs	Proximal Tubular Epithelial Cells
RNA	Ribonucleic acid
rRNA	Ribosomal ribonucleic acid
RT-PCR	Real Time-polymerase chain reaction
SDS	Sodium dodecyl sulphate
SDS-PAGE	SDS-polyacrylamide gel electrophoresis
S.E.M	Standard error of the mean
TGF-β1	Transforming growth factor beta 1
TIF	Tubulointerstitial fibrosis
U	Units
µg	Micrograms
µL	Microlitres
μM	Micromolar
v/v	Volume per Volume
